# A natural history of untreated chronic osteomyelitis of the tibia over 20 years, with evolving squamous cell carcinoma: a case report 

**DOI:** 10.5194/jbji-8-183-2023

**Published:** 2023-08-02

**Authors:** Asanka Wijendra, Alex Ramsden, Martin McNally

**Affiliations:** Bone Infection Unit, Nuffield Orthopaedic Centre, Oxford University Hospitals, Windmill Road, Oxford, OX3 7HE, UK

## Abstract

Squamous cell carcinoma (SCC) is a rare but potentially life-threatening complication of chronic osteomyelitis.

Whilst there have been over 100 cases of chronic osteomyelitis with malignant transformation reported in the literature between 1999 and 2020, this is the first case report to document transformation with 20 years of concordant imaging and clinical review.

## 1 Introduction

Osteomyelitis is an inflammatory and destructive condition of bone, secondary to infection. Haematogenous osteomyelitis is an infection which has been seeded through the blood stream, most commonly seen in prepubertal children and the elderly. Contiguous-focus osteomyelitis *without* vascular insufficiency occurs where there is either direct inoculation of the bone (such as following open fracture), or extension to bone from contaminated soft tissues. Contiguous-focus osteomyelitis *with* vascular insufficiency is almost exclusively seen in the lower limbs, most commonly as diabetic foot disease (Fritz and McDonald, 2008).

Acute disease can occur through any one of these routes, but the presence of dead bone marks the shift to chronic disease (McNally, 2021). A degree of devitalisation will occur at the time of injury, with the stripping of periosteum and intravascular thrombosis. This process can continue following the primary injury, with the activation of pro-inflammatory cell mediators and leucocytes resulting in increased intraosseous pressure, further impairing the circulation (Fritz and McDonald, 2008; McNally and Nagarajah, 2010).

The dead bone provides an avascular substrate to which planktonic bacteria can adhere (Barker et al., 2017; Fritz and McDonald, 2008; Chan et al., 2019). Irreversible adherence triggers modified gene expression within the bacterial colony, resulting in the production of exopolysaccharides (EPSs), water channels, and resultant biofilm formation (Sharma et al., 2019). Within these biofilms, the bacteria are better able to withstand changes in pH, phagocytosis, and the presence of natural and administered antimicrobials (Sharma et al., 2019). As a result, chronic sinus tracts often develop, delivering the dead bone (sequestra) along with pus to the skin surface (Corrigan et al., 2022; McNally and Nagarajah, 2010). The chronic nature of these tracts and presence of inflammatory mediators make the areas prone to ulceration and vulnerable to subsequent malignant transformation (Scanferla et al., 2022; Panteli et al., 2014; Pandey et al., 2009; Goldberg and Arbesfeld, 1991).

The relationship between chronic osteomyelitic ulcers and squamous carcinoma has been known for 170 years, and over 100 confirmed cases have been reported in the literature in the last 20 years (Sharma et al., 2011; Corrigan et al., 2022). However, the progression of the disease over many years has not been described in any case. In this report we describe a case of the malignant transformation of a chronic osteomyelitic ulcer with 20 years of concordant imaging and clinical review.

## 2 Case report

### 2.1 Background

**Figure 1:**
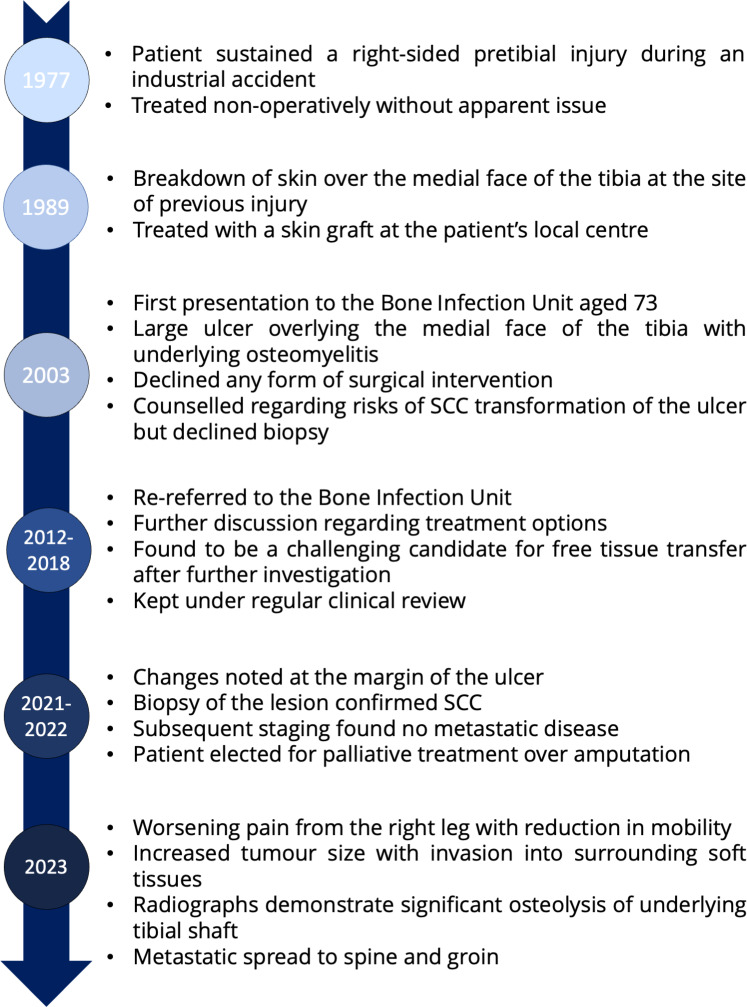
Timeline of patient history.

A 73-year-old man was referred to our Bone Infection Unit in 2003, with a discharging sinus from the anterior aspect of his right tibia. He had suffered a pretibial injury, following an industrial accident in 1977, aged 44. This was treated non-operatively, though he does recall being prescribed antibiotics by his primary care physician. In 1989 he reported that the skin overlying the site of injury broke down, for which he underwent debridement and skin grafting (Fig. 1).

**Figure 2:**
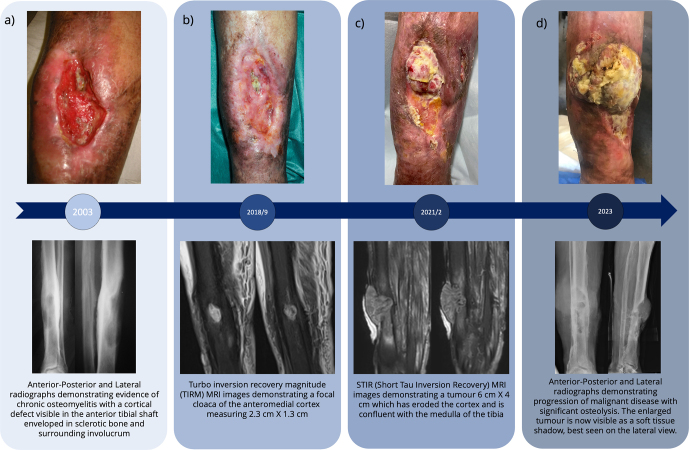
Clinical photographs of ulcer along with radiographs and MRI imaging from corresponding time points.

At first presentation to our unit, he had a large ulcer overlying the medial face of the tibia with underlying chronic osteomyelitis (Fig. 2a). Comorbidity factors included type 2 diabetes and obesity with a body mass index (BMI) of 42. He declined surgical intervention and was counselled regarding the risks of malignant transformation of the ulcer. He was offered a biopsy of the ulcer, but this was also declined.

He was re-referred in 2012, though once again he declined operative intervention, choosing instead to remain under regular review. In 2018, the patient decided to reconsider surgical intervention after noting increasing discharge from the ulcer. He underwent an MRI scan (Fig. 2b), ultrasound duplex scan, and vascular review. This revealed no imaging features of malignant transformation, but did identify a poor vascular supply beyond the trifurcation of his popliteal artery, rendering him a challenging candidate for free tissue transfer or local flap options. He therefore continued with regular dressings and yearly review.

In December 2021, it was noted that he had developed an exophytic lesion at the margin of his ulcer along with increasing malodour and discharge (Fig. 2c). Of note is that the lesion was not painful. It was biopsied and histological analysis identified squamous cell carcinoma. Subsequent staging workup revealed no evidence of metastatic disease. The patient was offered an amputation, which he declined and instead chose symptomatic control with his local palliative care team.

He underwent further review in March 2023, 14 months from initial diagnosis of malignant transformation. Over this period, he developed metastatic disease in the spine (without neurological compromise) and likely nodal metastases to the right groin. He reported that his mobility had reduced with increased reliance on a wheelchair. He remained otherwise systemically well, managing his symptoms with simple analgesia alone. His ulcer had grown (Fig. 2d), with ongoing malodour and discharge. This continues to be dressed twice weekly by his local community nursing team.

### 2.2 Imaging

As can be seen from the clinical photographs (Fig. 2), the ulcer appeared largely unchanged between 2003 and 2018. It had undergone short periods of almost complete healing, with marginal epithelialisation, followed by recurrent breakdown and enlargement. However, the ulcer did not fully heal at any point.

The photograph from 2021 demonstrates an exophytic mass which the patient reports originated at the superior margin of the ulcer and had gradually grown over the 6 months prior to review. By 2023 the exophytic mass has increased in size over the medial face of the tibia as well as infiltrating into previously healthy tissues within the lateral compartment of the leg.

**Figure 3:**
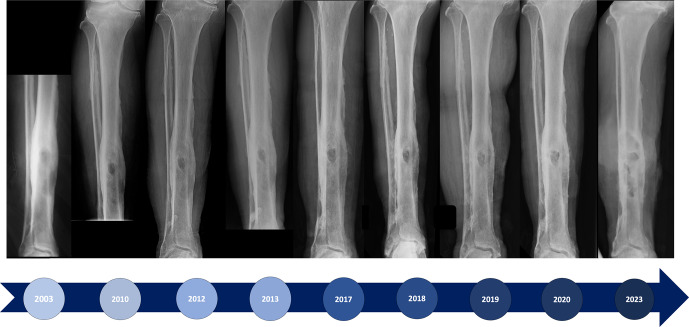
Anterior–posterior radiographs from 2003–2023.

**Figure 4:**
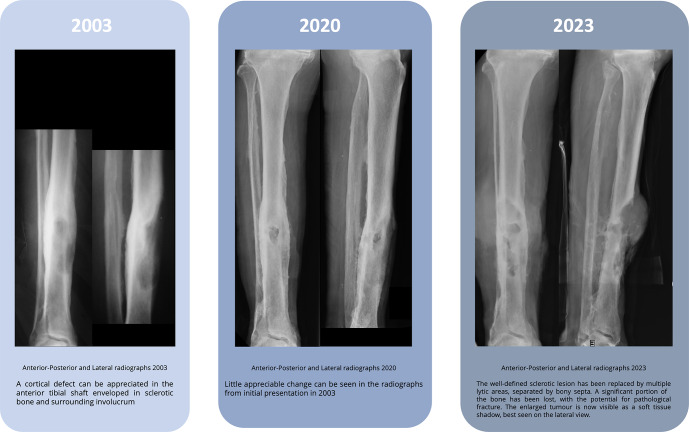
Anterior–posterior and lateral radiographs from 2003, 2020, and 2023.

Radiographs from 2003 demonstrate a cortical defect in the anterior tibial shaft enveloped in sclerotic bone and surrounding involucrum. There is little appreciable change in serial radiographs from initial presentation to the time of diagnosis of malignant transformation in 2021. However, by 2023 the well-defined sclerotic lesion is no longer visible. This has instead been replaced by multiple lytic areas, separated by bony septa, spanning approximately 10 cm of the tibia (Figs. 3 and 4). A significant portion of the bone has been lost, with the potential for pathological fracture.

An MRI in 2018 demonstrated a focal cloaca of the anteromedial cortex measuring 2.3 cm × 1.3 cm with debris in the cortex, likely representing multiple sequestra. There was a sinus tract communicating with the tibial medulla. By the beginning of 2022 the MRI showed a tumour, 6 cm × 4 cm, had replaced the subcutaneous fat overlying the anterior aspect of the tibia. This had eroded the cortex and was confluent with the medulla of the tibia with extensive endosteal scalloping (Fig. 2).

## 3 Discussion

Malignant transformation is a rare but serious complication of chronic osteomyelitis. The incidence has been reported to be between 1.6 % and 23 % (Panteli et al., 2014). The time between diagnosis of osteomyelitis and identification of malignant transformation is on average 31 years, though the range within the literature is wide (3–67 years) (Corrigan et al., 2022). While it is reported that it is often a late complication, with latent periods of over 20 years (Pandey et al., 2009; Panteli et al., 2014), it has been found to occur within much shorter time frames (Lack and McKinley, 2010; Corrigan et al., 2022). Indeed, in our unit there have been four cases identified since 1999 where malignant transformation occurred within 4 years of the onset of chronic osteomyelitis (Corrigan et al., 2022). It should therefore be suspected in all cases of chronic osteomyelitis, particularly those with a history of over 36 months with sinus formation (Corrigan et al., 2022). Squamous cell carcinoma is the most common type of malignant tumour identified (Corrigan et al., 2022; Goldberg and Arbesfeld, 1991; Li et al., 2015; Panteli et al., 2014), with the tibia being the most frequently affected site, followed by the femur (Corrigan et al., 2022; Li et al., 2015). Increased pain, malodour and discharge are the most common presenting symptoms of malignant transformation (Panteli et al., 2014).

Corrigan et al. (2022) investigated all cases of chronic osteomyelitis with malignant transformation reported in the literature between 1999 and 2020, including eight cases from our institution. Of the 106 cases identified, information regarding diagnosis was available for 49 cases. In 37 of these cases, a diagnostic biopsy prior to the planned definitive management identified the malignancy. In the remaining cases, diagnosis was made incidentally, following subsequent histopathological analysis of any excised or amputated specimens. Interestingly, the outcome was negatively associated with an incidental diagnosis rather than directed biopsy. It may be that in those cases of incidental diagnosis the condition had not been considered by the treating team and was not promptly investigated. Similarly, those patients who had metastatic malignant disease at the time of diagnosis had significantly poorer outcomes than those who did not (Corrigan et al., 2022).

**Figure 5:**
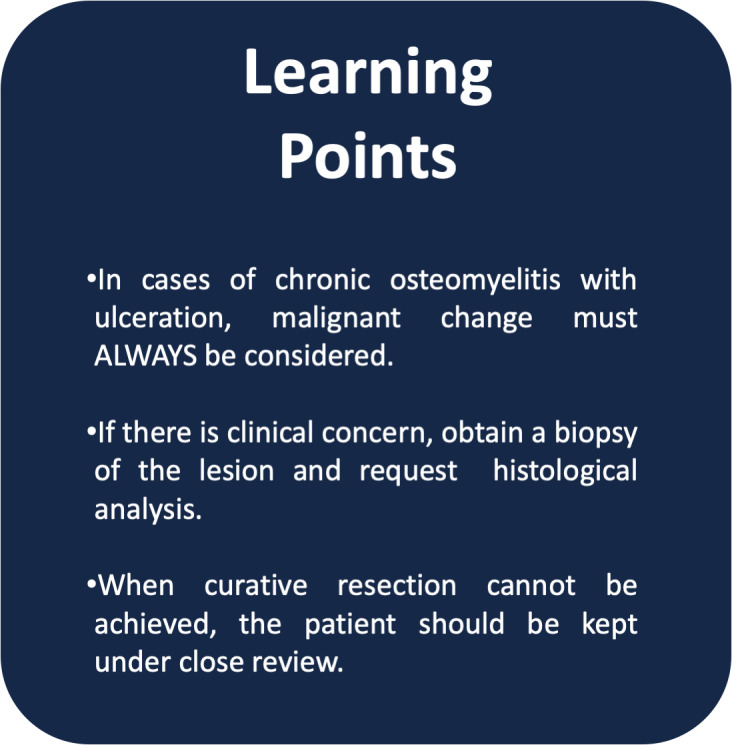
Learning points.

As with all malignancies, early detection and diagnosis is crucial to optimise outcome. As such, one must maintain a high level of clinical suspicion to diagnose malignant transformation promptly. This is especially important for patients where curative resection of the osteomyelitis has been ruled out, or declined, as a treatment option. In these cases, both the patient and their primary care practitioners must remain vigilant for signs of malignant transformation. They should be instructed that any cutaneous changes should be treated with a high index of suspicion and immediate referral for biopsy (Fig. 5).

Our patient exhibited the cycle of partial healing and recurrent breakdown which is typical of long-standing osteomyelitis. The mechanism for this is uncertain but may be due to the changes in the degree of inflammation around the ulcer. When the infection is very active, it will drain copious pus, compromise the surrounding skin, and extend the ulcer. After a period of increased activity, the immune response may be able to curtail the infection which will reduce the discharge. The skin can recover and may partially heal. However, further buildup of infection around the retained dead bone will restart the cycle of inflammation and increased ulceration. In our case, yearly review was instituted 3 years before the onset of malignancy. The clinical change was identified within a few months of onset and before metastatic spread had developed. This would have offered the possibility of local treatment.

If the biopsy is positive for malignancy, the patient should undergo systemic staging and review at an appropriate multi-disciplinary team (MDT) meeting, for consideration of adjuvant chemo or radiotherapy (Corrigan et al., 2022). Debate exists as to the optimum surgical management. Several studies have found amputation to be the surgical treatment of choice, particularly as this enables elimination of the chronic osteomyelitis along with the malignancy (Pandey et al., 2009; Panteli et al., 2014; Corrigan et al., 2022; Alami, 2011; Sağlik et al., 2001). However, successful outcomes have been reported following wide local excision where metastatic disease has been excluded (Ogawa et al., 2006). Further research in this area examining long-term outcomes following amputation versus excision is needed.

## Data Availability

Data are available upon request.
